# Support After Adolescent Behavioral Weight Loss Treatment During the Transition Into Emerging Adulthood: Qualitative Study

**DOI:** 10.2196/95649

**Published:** 2026-08-06

**Authors:** Laura Jean Caccavale, Katlyn Garr, Madison Weinstock, Maria D Thomson, Jessica Gokee LaRose, Kristina Tatum, Melanie K Bean

**Affiliations:** 1 Department of Pediatrics, Children's Hospital of Richmond at VCU, 2303 N Parham Rd Suite 1, Richmond, VA, 23298, United States, 1 804-527-4780; 2 Department of Psychiatry and Behavioral Sciences, University of Kansas Medical Center, Lawrence, KS, United States; 3 Department of Social and Behavioral Sciences, Virginia Commonwealth University, Richmond, VA, United States

**Keywords:** emerging adults, adolescents, parents, weight management, interviews, obesity

## Abstract

**Background:**

Obesity is a chronic disease requiring long-term treatment, yet current treatment models do not align with the chronicity of obesity. Current guidelines provide limited direction on how to adapt treatment to support weight loss maintenance as adolescents transition into emerging adulthood, a distinct developmental period from the late teens to the mid-to-late twenties.

**Objective:**

This qualitative study aims to (1) better understand adolescents’ and their parents’ experiences after behavioral weight-loss treatment as they navigate the transition from late adolescence into emerging adulthood and (2) identify the weight-management support needed during this transition. Formative data are critical to understanding the unique needs of youth with obesity after treatment, especially as they navigate the transition from late adolescence to emerging adulthood.

**Methods:**

Adolescents and parents (20 dyads; mean adolescent age 16.5, SD 0.6 y) completed semistructured interviews more than 11 months after participation in a 4-month multicomponent behavioral weight-loss treatment. Interview domains included adolescent and parent perceptions of facilitators and barriers to weight management following program completion, as well as needs and preferences for continued support during the transition to emerging adulthood. Adolescents were asked open-ended questions regarding their current health and weight management behaviors, perceived facilitators and barriers to engagement in health behaviors, parental and social support, and preferences for ongoing weight management support. Parents were asked parallel questions addressing their adolescents’ behaviors, their evolving roles as their teens transitioned to emerging adulthood, and recommendations for additional support. Interviews were recorded, transcribed verbatim, and thematically analyzed.

**Results:**

Adolescents and parents reported that adolescents maintained several weight management behaviors (eg, regular exercise, self-weighing, and improved diet quality), while identifying others as more difficult to sustain (eg, monitoring food intake and meal planning). Reported facilitators included consistent routines and various opportunities for physical activity (eg, diverse exercise options), while barriers included time constraints, competing priorities, and external influences (eg, the eating behaviors of other family members). Both adolescents and parents expressed a desire for continued support during the transition to emerging adulthood to sustain healthy behaviors and navigate changing roles. Most adolescents and parents expressed interest in participating in a booster program to support weight management during this period, particularly to enhance accountability. Preferences for frequency, duration, and format varied (eg, meeting frequency, program length, or in-person vs virtual).

**Conclusions:**

Findings identify key weight-management behaviors that families maintained after treatment, as well as areas that require ongoing support. Results underscore the need for continued support for weight-management behaviors during the transition to emerging adulthood and offer guidance on program structure and content. Results can be used to inform the development of a targeted behavioral weight-management transition program that addresses adolescents’ evolving needs during the shift to emerging adulthood, improving long-term outcomes and reducing health risks.

## Introduction

Approximately 21 million US adolescents and emerging adults, defined as individuals in their late teens through the mid-to-late twenties (aged 15‐24 y), are classified as having overweight (BMI>85th percentile) or obesity (BMI>95th percentile) [1,2]. Persistent racial, ethnic, and socioeconomic disparities are observed, with adolescents from racial or ethnic minority groups (ie, Black and Latine) and lower socioeconomic backgrounds at disproportionately high risk for disease [3]. Moreover, obesity rates increased during the COVID-19 pandemic and have since remained persistently elevated [4]. Obesity is a complex and multifactorial chronic disease that commonly persists into adulthood and is associated with significant lifelong morbidity and mortality [5-8]. Intensive health behavior and lifestyle treatment (IHBLT) is a first-line evidence-based behavioral intervention and the most effective known treatment [9]; however, current treatment models are generally short-term, resource-intensive, and not well aligned with the chronicity of obesity or the transition from adolescence to adulthood [10-12]. For example, only one adolescent trial has examined 2-year treatment effects, and because the sample was predominantly White [13], the findings may not generalize to groups experiencing health disparities. For some adolescents, management of obesity involves a multimodal approach, including medications and metabolic and bariatric surgery, but behavioral treatment continues to serve as a foundational part of care [14,15]. There is limited evidence regarding how adolescents fare after treatment or what support is needed as they transition into emerging adulthood.

Although modest short-term weight losses are typically observed in adolescent IHBLT [16], relapse is common, and to date, there are no established processes to support adolescents after treatment and to help them sustain weight losses, particularly as they transition into emerging adulthood. Indeed, a systematic review found “no evidence internationally of empirical research on the transition of adolescents with obesity from pediatric to adult weight management treatment,” and only a handful of clinical guidelines contained brief recommendations regarding this transition [17,18]. Current treatment approaches and guidelines do not align with the chronicity of obesity, highlighting an opportunity to better understand what support adolescents need as they transition into emerging adulthood. A recent study emphasizes that, without adequate support, adolescents are at risk of losing continuity in health care, which may increase the likelihood of complications [18]. The clinical practice guideline from the American Academy of Pediatrics underscores the importance of a structured, individualized care plan for adolescents, including coordinated collaboration between primary care providers, pediatric specialists, and the adult health care professionals who will assume their care as they transition into adulthood [9]. There is a need to determine how to best stage interventions in the context of chronic care and to identify what posttreatment supports are needed to mitigate health risks during the transition into emerging adulthood [19]. Furthermore, given the unique developmental stage of emerging adulthood [2,20], it is important to understand how to prepare adolescents to manage their health in this transitional life stage.

Emerging adulthood is marked by instability and multiple life transitions across physical and interpersonal environments [2,20,21]. The need for independence increases and reliance on parents and caregivers decreases, as adolescents assume primary responsibility for their health while also navigating these life transitions [22]. Numerous unhealthy weight-related behaviors [23-29] (eg, alcohol use and high levels of sedentary behavior) and disordered eating behaviors (eg, binge eating) peak during these years, and significant weight gains are commonly observed, with the largest gains among those with overweight or obesity [30,31]. Alarmingly, more than 40% of emerging adults have overweight or obesity [32] and continuous obesity from adolescence to emerging adulthood intensifies health risks [33]. Despite the need, emerging adults are vastly underrepresented in adult IHBLT trials [34], clinical settings [35], and commercial weight management programs [36]. Further, very few IHBLT trials have been designed for this age group specifically [37-39], and to our knowledge, no programs have been designed to support adolescents in maintaining weight loss during this developmental transition.

In sum, formative data are essential for understanding the unique needs of youth with obesity after treatment and as they traverse late adolescence through emerging adulthood. Thus, this study, TEENS Talk, conducted qualitative interviews with older adolescents (aged 16‐18 y) and their parents who recently completed an IHBLT program to understand adolescents’ and their parents’ experiences after behavioral weight loss treatment as they navigate late adolescence into emerging adulthood and to identify what weight management support is needed during this transition. Domains assessed included: (1) weight management skills maintained or discontinued, (2) facilitators and barriers to continued weight management, (3) skills needed to navigate this transition into emerging adulthood, and (4) ideas for a booster program to support this transition.

## Methods

### Participants and Recruitment

Participants in the TEENS Talk study (N=20) were a subset of adolescent (aged 16‐18 y) and parent or caregiver dyads who completed the TEENS+ randomized clinical trial, a 4-month multicomponent IHBLT [40]. Parent adolescent dyads were randomized to one of two 4-month treatments in which parents were either engaged as helpers in their child’s weight management via parent skills training based on authoritative parenting or engaged in their own behavioral weight management. All adolescents received nutrition education with dietary goals, supervised physical activity, and behavioral support [40]. Families were eligible for TEENS Talk after completing the final 12-month follow-up assessment for TEENS+. During recruitment for this study, 41 TEENS+ families were eligible for participation, and 40 were approached (1 was not approached due to a parent moving out of the country and being unable to be contacted). Of those who were eligible, 5 declined and 15 were unresponsive to contact attempts or lost to follow-up. Exclusion criteria for the TEENS+ trial included type 2 diabetes and a history of metabolic and bariatric surgery. Detailed methods and procedures for the TEENS+ trial may be found elsewhere [40]. Following their 12-month follow-up assessments, TEENS+ participants were contacted by study staff via email and telephone and invited to participate in this qualitative study, with recruitment continuing until the target sample size was reached. The target sample size was informed by prior research and selected to ensure adequate data for achieving thematic saturation [41].

### Study Procedures

Trained interviewers conducted semistructured interviews via Zoom (Zoom Communications, Inc), with separate interviews for adolescents and parents. Interviews were approximately 50 minutes in duration (range 29-85 min for adolescent interviews; range 26-71 min for parent interviews). Adolescent participants were asked open-ended questions about their current health and weight management behaviors (eg, “What, if any, weight management behaviors are you working on now?”), perceived facilitators and barriers to engagement in health behaviors (eg, “What helps you keep up with weight management behaviors?”), the parental role and other support in health behaviors (eg, “What, if any, role do your parents play in supporting your weight management behaviors?”), and preferences for continued weight management support during the transition into emerging adulthood (eg, “Thinking about getting older and becoming a young adult, potentially living apart from your family, what do you think will help you set up your environment to make you successful with weight management behaviors?*”*). Parents were asked corresponding questions about their adolescents’ weight management behaviors, the parental role as their teen transitions to emerging adulthood (eg, “As your teen transitions from adolescence to young adulthood, how might your role providing support for weight management change?*”*), and ideas for additional support (eg, “Would you be interested in support as part of a specifically focused on teaching you strategies and how to provide support to your adolescent/young adult during this transition? If so, what topics would like to be covered?*”*). Interviews were audio recorded, transcribed verbatim, and checked for accuracy. The SRQR (Standards for Reporting Qualitative Research) guidelines (Checklist 1) [42] were followed for this manuscript.

### Ethical Considerations

All study procedures were approved by the Virginia Commonwealth Institutional Review Board (HM20022326). For parents and adolescents aged 18 years or older, written consent was obtained. For adolescents younger than 18 years of age, parental permission was obtained and adolescents provided verbal assent. All data in this study were deidentified before the coding process. Participating families were provided with a US $50 gift card, which was given to the parent, as compensation for their time.

### Data Analysis

A modified grounded theory approach combined with thematic analysis was used to guide qualitative coding [41], with the goal of identifying key themes and generating insights to inform future intervention development. Transcripts were reviewed and coded by the coding team, comprising a clinical psychologist (LC), postdoctoral fellows (KG and KT), and a graduate doctoral student (MW). Specifically, coding and theme development were conducted iteratively, beginning with open coding to capture emerging ideas, followed by grouping similar codes into broader themes. Themes were reviewed and refined through ongoing comparison within and between interviews. The coding framework included categories (eg, skills maintained, skills discontinued, facilitators, and barriers), codes, subcodes (when appropriate), and descriptions of codes and examples (when appropriate). The coding framework was updated as needed through team discussions to ensure clarity and consistency.

ATLAS.ti Qualitative Data Analysis Software (version 23) was used to apply codes to segments of the transcripts. Coders independently double-coded a subset of transcripts (20%, or 4 transcripts), which yielded high coder agreement (intraclass correlation coefficient>0.80), suggesting that independent coders were consistently applying the same codes to the same transcripts. The coding team then independently analyzed transcripts for adolescents and parents (separately). Any ongoing discrepancies in coding were discussed until resolved during weekly team meetings, and all disagreements were fully resolved through discussion. To enhance reflexivity and minimize bias, the team engaged in regular debriefings throughout data analysis to examine assumptions, question interpretations, and ensure that participant voices guided theme development. To enhance trustworthiness, multiple researchers independently reviewed and verified the findings. All transcripts and versions of the codebook were saved as separate documents to create an audit trail of changes.

## Results

### Sample Characteristics

The average adolescent age was 16.5 (SD 0.6) years and parent age was 48.4 (SD 6.9) years. The average BMI for adolescents at baseline in the TEENS+ trial was 34.98 (SD 6.5) kg/m^2^. The average adolescent age of TEENS+ participants when they completed the IHBLT TEENS+ program was 14.8 (SD 1.11) years, with a range of 11 months to 2 years and 11 months since active treatment. See Table 1 for participant demographics. Overall themes identified were related to engagement in weight management behaviors (eg, skills maintained and discontinued, barriers and facilitators to weight management behaviors) and the support needed to engage in behavioral weight management during the transition to emerging adulthood.

**Table 1.** Participant demographics (N=20).

Characteristics	Adolescents	Parents
Sex, n (%)		
Male	11 (55)	4 (20)
Female	9 (45)	16 (80)
Age (y), mean (SD)	16.5 (0.6)	48.4 (6.9)
BMI (kg/m^2^), mean (SD)		
Baseline BMI from TEENS+ trial	34.98 (6.5)	—^a^
Race, n (%)		
White	10 (50)	11 (55)
Black or African American	7 (35)	6 (30)
Asian	1 (5)	1 (5)
Multiracial	1 (5)	0 (0)
Other	1 (5)	2 (10)
Ethnicity, n (%)		
Hispanic or Latino	1 (5)	1 (5)
Not Hispanic or Latino	19 (95)	19 (95)

a Not applicable.

### Engagement in Weight Management Behaviors

Adolescents and parents described progress in adolescent weight management following the 4-month TEENS+ program. Subthemes emerged in the following categories related to engagement in weight management behaviors: (1) skills maintained, (2) skills discontinued, (3) facilitators of engagement in weight management, and (4) barriers to engagement in weight management.

### Skills Maintained

Adolescents and parents described a variety of weight management skills, including exercise, nutrition, and self-regulation, that were maintained following the 4-month IHBLT treatment. The 5 most frequently maintained behaviors described by adolescents were: exercise, portion control, self-regulation or self-weighing, improved diet quality (eg, eating more healthy foods), and nutrition swaps. One adolescent described the importance of fitting exercise into their daily routine:

*Yeah so definitely after the TEENS program, I’ve tried to incorporate exercise more into my life, even if there’s like 15 minutes or 5 minutes. I just try to do I something*

[Teen ID 11, 17 y, female, other race]

Adolescents and parents also described differences in maintaining weight-management behaviors, such that adolescents frequently noted that portion control was a behavior they had maintained, whereas parents commonly reported maintaining mindful choices about what to eat or buy. A parent described how they make mindful choices for their family: “We are definitely more aware and really just trying to stay away from the processed foods” (Parent ID 04, 48 y, female, African American or Black). See Table 2 for additional sample quotes.

**Table 2.** Example quotes from adolescents and parents related to weight management skills that were maintained and discontinued following a behavioral weight-loss program^a^.

Domain	Adolescent	Parent
Skills maintained		
Exercise	“I do a lot of just movement as much as I can, especially at school, my school is three stories tall, so we have a lot of stairs that. We have to go up and down and I’m in a mentorship right now is one of my classes, and that is like a block or two away from my school, so I walk over there” (Teen ID 03, 17 y, female, White).	“He (TEEN) is walking to school every day, so that’s five days a week. It’s about a mile and a half walk home. He has joined wrestling workouts, so offseason wrestling workouts” (Parent ID 17, 55 y, male, White).
Nutrition	“If I do eat sauces like mayo or ranch, I always eat light ranch or light mayo” (Teen ID 02, 16 y, male, White).	"Not so much processed foods or fast foods more like eating from home, home cooked meals watching sodium, sugar, you know stuff like that” (Parent ID 08, 65 y, female, White).
Self-regulation	“What I found is a Google spreadsheet to keep track of my food logs. So, I moved to doing a spreadsheet and I can see when I entered the data, so I can chart it and I can see where I am” (Teen ID 05, 16 y, female, White).	“I remember one time she made a smoothie, and then I was like, “Do you want me to order some Boba tea?” and she was like “Oh no, I can’t because I already had a smoothie,” So, I do see her just being more conscientious of her choices” (Parent ID 11, 39 y, female, other race).
Skills discontinued		
Exercise	“I can’t remember the last time I worked out, I’m gonna [be] honest. I was doing real good with the working out, like I really was. And the crazy part about it is, I even felt better like when I worked out because obviously why wouldn’t you?” (Teen ID 01, 17 y, female, African American or Black).	“I think that [TEEN] is not moving. I think that she has gotten herself a little bubble in the living room where she has a study station [when] they were virtual, and that has become her place. She is either there or in her room” (Parent ID 05, 53 y, female, White).
Nutrition	“I did some meal planning, like I wrote down what I was going to eat for breakfast, what I was going to eat for lunch, and what I was going to eat for dinner. But after I left TEENS I kind of stopped doing it a little more, and I don’t do it as much” (Teen ID 20, 16 y, male, White).	“Yeah, I’m not really managing his food, and even at dinner I’m not as good at it, I’m not cooking as good of meals. It’s always easier for myself to manage my lunch, because I’m away from the house. Dinners - they’re not great, they’re just too much work” (Parent ID 16, 49 y, female, White).
Self-regulation	“I don’t weigh myself very often. Pretty much the only time I do is when I, like, go to the doctor” (Teen ID 15, 16 y, male, White).	“We were recording everything we ate, we were using the app, you know, logging everything. Yeah, that was kind of hard, and I don’t do that anymore” (Parent ID 07, 55 y, female, White).

a The ”Exercise” domain includes descriptions of exercise and lifestyle physical activity. The “Nutrition” domain includes descriptions of diet quality, nutrition swaps, balanced meals, and meal planning. The “Self-Regulation” domain includes descriptions of self-weighing, logging or monitoring food intake, and portion control.

### Skills Discontinued

Adolescents and parents also described discontinuing certain weight management behaviors following IHBLT treatment. The most common behavior that both adolescents and parents reported had ended was monitoring food intake (eg, logging or tracking food and counting calories). For example, adolescents described forgetfulness and low motivation as barriers to monitoring their food intake:

*I didn’t really do logging that well, because I am very ADHD, and I just forget to do stuff.*

[Teen ID 14, 16 y, male, Multiracial]

*She [mom] tried to get me to log my food, but I just couldn’t after because I was too lazy to do that.*

[Teen ID 02, 16 y, male, White]

Other commonly discontinued skills reported by both adolescents and parents were exercise, self-weighing or self-regulation, and meal planning or preparation. Parents also described portion control as a skill that was difficult for their adolescents to maintain after the IHBLT program. One parent noted:

*I think it’s a volume more than the choice of food that he’s eating so he’ll eat a whole bunch of you know, like a whole package of frozen mango or something like that which is reasonably healthy thing, but when it’s in a bag that’s this big, it’s the amount of it that that could potentially be problematic.*

[Parent ID 07, 55 y, female, White]

See Table 2 for additional sample quotes.

### Adolescents Found Internal and External Support Useful in Sustaining Weight Management Behaviors

**Figure 1.**
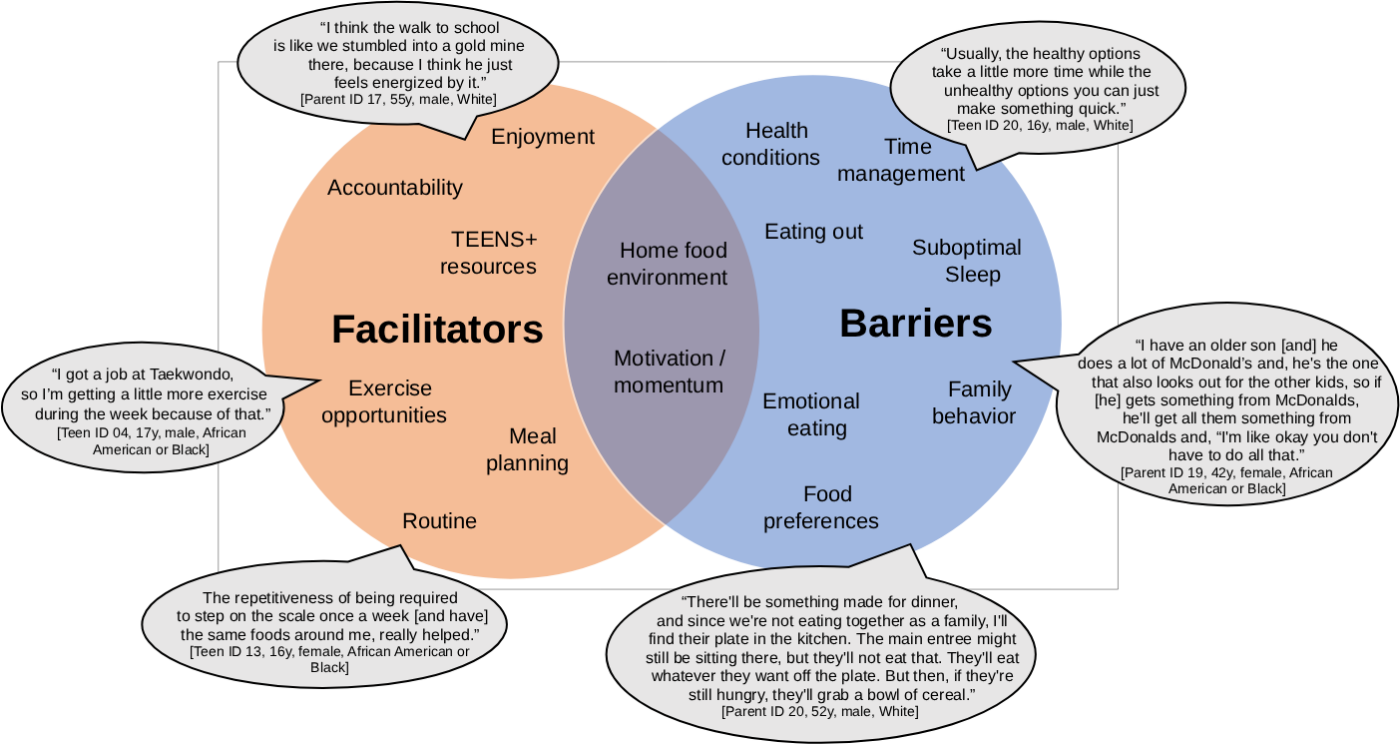
Adolescent and parent facilitators and barriers to weight management.

Adolescents and parents described several factors that facilitated ongoing weight management behaviors in Figure 1. For example, both highlighted the value of maintaining a consistent routine and having multiple opportunities for physical activity (eg, a yoga mat, a treadmill, or a gym membership). One adolescent emphasized how simplicity and consistency supported healthy eating:

*I don’t eat a bunch of different stuff so it’s the same 10 meals. And I’m just pretty consistent with that, we cook the same stuff*

[Teen ID 07, 17 y, male, White]

Many parents described the intervention resources and materials as valuable supports for maintaining their own weight management behaviors and assisting their adolescent’s ongoing efforts. For example, “I think the TEENS program had given him the resources to make the decisions and [help him] practice the healthy lifestyle” (Parent ID 04, 48 y, female, African American or Black).

Adolescents also noted that their environment, including their home food environment, served as facilitators for their weight management. One adolescent described the importance of healthy food being easily available, “We have fruit sitting around the counter, so when you walk in you see them” (Teen ID 06, 16 y, female, African American or Black).

### Competing Demands Impeded Adolescents’ Engagement in Weight-Management Behaviors

Adolescents and parents highlighted a range of challenges that hindered their ability to sustain weight management behaviors following the intervention, as shown in Figure 1. Prioritizing health behaviors and time management were frequently described as barriers to weight management for both adolescents and parents. For example, 1 adolescent described the issue of competing responsibilities as a barrier to meal preparation:

*For meal prep, it’s not like my biggest priority in the nighttime, my biggest priority is take care of myself like taking a shower, and getting ready for the next morning and do my homework, or if my mom needs help with something like I have to help my brother shower, or like help her clean or something. And you know if I don’t have the time to meal prep the night before then I have the morning but it’s usually a rush*

[Teen ID 11, 17 y, female, other race]

Other frequently described barriers for adolescents and parents include environmental factors (eg, working in fast-food service) and other family members’ behaviors (eg, parents or siblings are not focused on weight management). Parents also noted that adolescent food preferences, motivation, and energy were barriers. One parent stated, “I’ve been looking at menus because he’s a very picky eater, doesn’t like to try new things…so it’s kind of hard for me to find a new meal to offer up” (Parent ID 08, 65 y, female, White).

### Preparing for the Transition to Emerging Adulthood

Adolescents and parents both anticipated numerous changes in weight management approaches as adolescents transitioned into emerging adulthood. Three subthemes emerged regarding perceived facilitators of weight management success in emerging adulthood: (1) growing autonomy, (2) continued development of relevant skills, and (3) reliance on peer and parent support.

### Growing Autonomy

Adolescents and their parents identified that the transition to emerging adulthood would require increased independence. Many adolescents expressed excitement over increases in their independence. For example, 1 adolescent noted that they were, “Really grateful that it is time to graduate [from high school] and move on, because it can give me more freedom” (Teen ID 03, 17 y, female, White). Other adolescents anticipated having some challenges associated with increased autonomy, with 1 adolescent describing, “I think my main worry is when you go to college…I have to make a lot of food decisions by myself*”* (Teen ID 12, 16 y, female, Asian). Parents also considered the impact of increases in their adolescents’ independence:

*I won’t have very much control at that point. I won’t have any, actually…They’re adults, we have to trust them that they’ll make good decisions*

[Parent ID 05, 54 y, female, White]

Parents expressed hopefulness that their adolescent would use this independence effectively, and that they would “Transfer these skills when I’m not around*”* (Parent ID 04, 48 y, female, African American or Black).

### Continued Development of Relevant Skills

Adolescents and parents identified skills that would be necessary for an effective transition. They anticipated maintaining many of the skills they already possessed, including exercising, making healthy food choices, and maintaining healthy routines. One adolescent noted that he would need to “Just [keep] doing the same things I do now” (Teen ID 14, 16 y, male, Multiracial). Adolescents and parents also realized that there were multiple new skills they would have to learn. Participants recognized that the adolescent would need to take on some of the roles that the parent had previously held. For instance, 1 adolescent said, “The grocery thing was something I hadn’t thought of because that was something that my mom had learned about with the program, but that wasn’t a thing that stuck with me because it wasn’t relevant to me at the time” (Teen ID 07, 17 y, male, White). Adolescents also realized they would need to develop additional skills, such as time management and eating healthy while eating out, that would be particularly relevant to their life stage. For example, 1 adolescent said:

*I’m not too sure what food is like in college. Like, if I can cook for myself in a dorm room, or if you have to go to a dining hall. But whatever the option may be, I just have to be on top of it—to see what the options are and then make the choice of what to eat*.

[Teen ID 13, 16 y, female, African American or Black]

### Reliance on Peer and Parental Support

Although adolescents and parents both recognized a shift toward increased independence, they also recognized the importance of continued support from peers and family. Adolescents recognized that they would need support from “Friends that are doing the same thing” (Teen ID 19, 16 y, male, African American or Black) to ease their transition. They also anticipated needing support from family members. Adolescents hoped that their parents would take on more of a role in checking in or providing reminders. For instance, when asked how their parent could help, 1 teen stated, “They can just remind me to do it, encourage me to do it*”* (Teen ID 16, 16 y, male, White). Parents were also actively considering how their role might change. They acknowledged that they would continue to support their adolescent, but that*:*

*The role becomes much more arm’s length. And kind of coach—asking questions gently and offering advice or feedback*.

[Parent ID 07, 55 y, female, White]

Rather than providing instrumental support, like meal preparation and grocery shopping, they anticipated focusing on emotional support and reminders. One parent described their role as, “We have to make them feel like we’re here, we’re their safety net, we’re cheering them on, we’re always going to be in their corner*”* (Parent ID 05, 53 y, female, White).

### Ideas for a Booster Intervention for Emerging Adults

Participants were asked about their interest in receiving support during emerging adulthood from a booster IHBLT program specifically designed to help them manage health goals as they transition into emerging adulthood. Most adolescent participants were interested in a booster program (n=13, 65% interested; n=5, 25% maybe interested; and n=2, 10% not interested). Participants described the topics they would like to address and their preferences for the structure of a potential booster program. See Table 3 for details.

Preferences for a booster program varied. Both parents and adolescents said that group accountability would be useful. Multiple adolescents expressed interest in including incentives, such as prizes, in the program. Both adolescents and parents identified opportunities for group exercise as being of great interest to them. Parents and adolescents also requested more cooking classes, which were a component of the original TEENS+ IHBLT program. Parents expressed interest in their adolescents having a professional to speak with about weight management, as they believed their adolescents were able to hear feedback from a professional more easily than from their parents. Preferences for the frequency and duration of sessions varied considerably (ie, group meetings 3 times a week or once a month and a program lasting 1 mo or 6 mo), as did the desire for in-person versus virtual sessions.

**Table 3.** Potential topics for a behavioral weight-management booster program.

Topics	Adolescents	Parents
Recipes	“Learning more quick recipes you can cook at home that actually taste good and maybe include a couple of GO foods” (Teen ID 18, 16 y, female, African American or Black)	“More meatless things, more snacks I could have available that are low calorie, smoothie recipes, stuff like that” (Parent ID 18, 45 y, African American or Black)
Meal planning	“Really just meal planning and getting better at grocery shopping” [Teen ID 15, 16 y, male, White).	“Ideas of what she can eat while she’s on college campus” (Parent ID 10, 35 y, female, African American or Black)
Nutrition choices	“What good foods to eat and what types of foods are the best for me” (Teen ID 20, 16 y, male, White)	“Finding healthier alternatives to the things he likes” (Parent ID 19, 42 y, female, African American or Black)
Exercise	“Going back over exercise and the ways I can exercise” (Teen ID 02, 16 y, male, White)	“The impact of exercise, that’s the main thing” (Parent ID 09, 50 y, male, White)
Mood and mental health	“For people who eat when they’re stressed, maybe some ways to de-stress” (Teen ID 13, 16 y, female, African American or Black).	“Her anxiety feeds her desire to eat. So I think addressing how it all fits together would be helpful” (Parent ID 05, 53 y, female, White).
Body image and relationship with food	“Topics that people don’t normally talk about—like body image, especially how social media influences our mental health and eating habits” (Teen ID 11, 17 y, female, other race)	—^a^
Parent-child communication	—	“I think there is definitely an opportunity to learn how to interact, and how to encourage without challenging. Or how to encourage without pushing them away” (Parent ID 17, 55 y, male, White).

a Not applicable.

## Discussion

This study examined adolescents’ and parents’ perspectives following behavioral weight loss treatment to gain insight into their experiences and identify ongoing support needs for sustained weight management during the transition to emerging adulthood. Adolescents and parents described weight management behaviors that were successfully maintained and those that were more difficult to sustain following the 4-month IHBLT program. Regular exercise, self-weighing, and improved diet quality were commonly maintained behaviors, whereas monitoring food intake and meal preparation were described as more challenging to continue. Participants emphasized the importance of increased autonomy, continued skills development, and external support to facilitate successful weight management during the transition from adolescence to adulthood. Most adolescents expressed interest in a booster program and offered specific ideas for program content, highlighting the need for ongoing, developmentally adapted interventions for this high-risk population.

Understanding which weight management behaviors are maintained or discontinued following IHBLT programs is critical for refining and optimizing intervention strategies, particularly given the chronic course of adolescent obesity and its associated comorbidities [5-7]. In this study, behaviors such as engaging in regular exercise, eating healthier foods, and self-weighing were among the easier skills for adolescents to maintain. This finding is consistent with prior research indicating that skills such as exercise and self-monitoring are often perceived as more feasible or rewarding to maintain [43,44]. Identifying such behaviors can help determine which components should be emphasized and reinforced throughout treatment. In addition, identifying behaviors that are more difficult to maintain (eg, logging food intake and meal planning) provides valuable insight into areas that may require additional support during and following treatment. Importantly, there was overlap between maintained and discontinued behaviors, highlighting the need to account for individual differences and to tailor programs accordingly. Similarly, understanding key facilitators (eg, consistency and accessible resources) and barriers (eg, time management and competing priorities) from both adolescent and parent perspectives allows for targeted adaptations to enhance engagement and long-term adherence. Together, these insights can guide the development of intervention strategies to promote sustained weight loss following initial treatment during adolescence, such as booster sessions, peer support, or continued experiential learning.

Consistent with prior research, both adolescents and parents emphasized the need for developmentally adapted approaches that address the unique challenges and increasing independence characteristic of this life stage [45]. Effective weight management requires interventions that are specifically adapted and designed accordingly. Broad themes that emerged from these data highlight adolescents’ growing autonomy, the need for adolescents to develop new skills as they enter emerging adulthood, and the importance of external support as important considerations relevant to weight management for this population. Supporting families in navigating the transition to emerging adulthood requires fostering adolescents’ autonomy while maintaining developmentally appropriate parental involvement. Models of health care transition for various chronic conditions emphasize the gradual development of adolescent independence alongside continued, appropriate parental support [46,47]. Our findings underscore the need for interventions that intentionally foster autonomy in adolescents engaged in weight management, emphasizing a gradual shift toward independent self-management as they transition into emerging adulthood.

Participants emphasized the importance of both peers and parents in supporting weight management behaviors during the transition to emerging adulthood. While the influence of peers on weight-related health behaviors during this developmental period is well-established [48,49], the role of parents is less clearly understood. Recent research examining discussions between emerging adults and their parents around weight and health suggests that frequent weight-focused conversations are associated with negative health outcomes, whereas less frequent, health-focused discussions are more likely to be linked to positive outcomes [50]. The finding regarding the important role of peers in this population is consistent with prior research. For example, among adolescents undergoing metabolic and bariatric surgery, social support has been identified as a key factor in enhancing motivation and engagement [51]. Providing support and education to parents or caregivers of emerging adults engaged in weight management remains understudied but may be critical in helping this population achieve and sustain their health and weight-related goals.

Given that obesity is a chronic health condition, long-term treatment models are needed to support individuals with obesity throughout the lifespan, especially as they transition into new developmental stages. The transition into adulthood is a particularly high-risk time for weight gain among individuals with obesity [32], highlighting the necessity for treatments designed to attenuate this risk. Participants in this study identified a variety of anticipated challenges associated with transitioning into early adulthood, and programming that supports that transition was of interest to most adolescents and parents. Adolescents expressed a desire to review information they learned in the original program, learn new skills necessary for their upcoming life stage, and discuss other relevant topics not introduced in the original program (ie, body image and stress management). Parents similarly desired a review of skills and requested information on how to effectively communicate with their adolescents about weight management. The findings of this study underscore the need to develop a booster program for adolescents and parents who have previously participated in behavioral weight loss treatment and provide valuable insights into participant preferences for such a program. Additionally, results support the idea of a chronic care model for obesity management, integrating prevention and intervention strategies at key developmental stages, including the transition to emerging adulthood. Overall, both adolescents and their parents expressed a desire for continued support following program completion. Findings suggest that adolescents and their families may benefit from structured support, including peer and social support components, within a broader chronic care and transitional care framework.

Although this study provides important perspectives from both adolescents and parents, its findings are constrained by a focus on adolescents who have completed an IHBLT program, potentially limiting generalizability to other populations. However, as access to IHBLT expands, an increasing number of adolescents with obesity may complete such programs and subsequently seek additional support to sustain their progress. Furthermore, variability in the timing of posttreatment interviews (ranging from 11 mo to 2 y and 11 mo) may affect recall, how behaviors are maintained, and the perceived need for booster support. Data interpretation in qualitative research may be influenced by coder bias; however, the use of team-based coding and consensus discussions helps to minimize this limitation. The inclusion of both adolescent and parent perspectives, and the representation of adolescents from diverse racial backgrounds are strengths of this study. Another limitation is that participants were drawn from families who completed the TEENS+ trial, likely reflecting those who are more engaged, satisfied, or successful, and whose support needs may differ from those of nonparticipants.

In sum, despite recognition that adolescent obesity is a chronic condition requiring long-term management, current treatment approaches remain largely short-term and guidelines remain vague regarding how treatment should evolve. Indeed, there are no evidence-based treatment models to support adolescents following treatment as they transition through emerging adulthood. Findings from this qualitative study provide valuable insight into what additional support may be needed to sustain and facilitate weight management behaviors among adolescents following behavioral weight loss treatment and as they transition into emerging adulthood. These findings have the potential to advance scientific understanding and clinical care by informing the development of a chronic care framework that accounts for the evolving needs of adolescents as they transition into emerging adulthood.

## Supplementary material

10.2196/95649 Checklist 1 SRQR checklist

## Abbreviations

IHBLT: intensive health behavior and lifestyle treatment
SRQR: Standards for Reporting Qualitative Research
